# Supplementary material for: Transition from light diffusion to localization in three-dimensional amorphous dielectric networks near the band edge
Source: Nat Commun. 2020 Sep 25;11:4867. doi: 10.1038/s41467-020-18571-w (PMC7519077; doi:10.1038/s41467-020-18571-w)
Supplement: Supplementary file 1 — Supplementary Information [file 41467_2020_18571_MOESM1_ESM.pdf]

Supplementary Information: “Transition from Light Diffusion to Localization in Three-Dimensional Amorphous Dielectric Networks near the Band Edge”

Jakub Haberko,[1*] Luis S. Froufe-Pérez,[2*] Frank Scheffold,[2†]

[1]Faculty of Physics and Applied Computer Science, AGH University of Science and Technology, al. Mickiewicza 30, 30-059 Krakow, Poland

[2]Department of Physics, University of Fribourg, 1700 Fribourg, Switzerland

[*] These authors contributed equally to this work.

[†]To whom correspondence should be addressed; E-mail: frank.scheffold@unifr.ch.

Supplementary Note 1: Evanescent wave tunneling around the gap center frequency ν'_{Gap}

Over a range of frequencies around ν'_{Gap} the transmittance $T(L/a, \nu')$ decays very rapidly, over the length scale of $L_B \sim a$, as shown in Fig. 1(d). We identify the center of this region as the photonic band gap (PBG), in agreement with the DOS calculations shown in Figure 1(b). To extract the decay length L_B we perform a linear fit to the first eight points $\ln(L/a)$ with $L/a \in [0.3, 2.4]$ as shown in Fig.1 (d). In Supplementary Figure 1 we show the corresponding error S of the least-squares fit. We find that initial exponential decay for frequencies $\nu' \in [0.46, 0.495]$

We note that for $T < 10^{-2}$ we observe deviations from a single exponential 'evanescent' decay. With $L_B/a \simeq 0.75$ at $\nu' = 0.48$ we would expect $T < 10^{-3}$ for $L/a \geq 5.5$. Our numerical data, shown for example in Fig. 6(d), suggests $T > 10^{-3}$ up to $L/a \simeq 10$. We tentatively explain this increased transmittance with the presence of spurious defect states at the boundary. When constructing the slab geometry with periodic boundary conditions, as discussed in the text, we expect a slight mismatch. This mismatch is due to the (to us) unknown exact size of the simulation box for the seed pattern, originally taken from reference (*1*). For the band gap calculations using MPB shown in Figure 1, we had to generate new and smaller disordered packings in order to match the available computational resources. To this end we were using the code provided by Torquato and coworkers upon request and described in (*2*). Here, the size of the simulation box is precisely known and the DOS in the gap is exactly zero. We note that in earlier work, we have observed that a slight mismatch of boundary conditions indeed leads to defect states in the band gap (*3*).

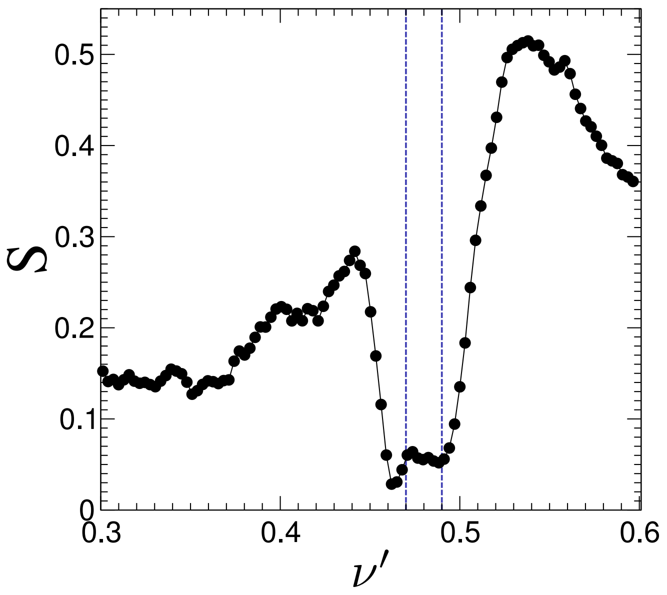

Supplementary Figure 1: Fitting error S (least-squares) for a linear fit of $\ln T(L)$ over $L/a \in [0.3, 2.4]$ (N=8 points). The initial exponential decay is observed for frequencies in the gap $\nu' \in [0.46, 0.49]$ and near the band edges. The vertical lines mark the band edges from MPB, as reported in Figure 1(b).

Supplementary Methods

Density of states and comparison to results by Liew et al. Practically identical network structures have been studied by Liew et al. in a recent study of the optical DOS (*4*). They report a strong depletion of the DOS, by more than two orders of magnitude, over a significant range of frequencies, indicating the presence of a band gap. Moreover, they show that networks with a filling fraction of $\phi = 0.28$, display the most pronounced photonic properties (*4*). In Supplementary Figure 2 we compare our band structure calculations, using the supercell method implemented in MPB (*5*), to their results using a different algorithm. We are not familiar with the details of their numerical method but apparently there are limitations in accuracy or convergence which lead to a finite density of states even in the gap center. Our results, using the established supercell method, with periodic boundary conditions, confirms the existence of a

full band gap (*3, 5, 6*).

More specifically, we start by considering a cubic lattice of lattice parameter L. In order to avoid the emergence of surface states due to the non conformity of the periodic cell with the actual structure, we consider a lattice basis which is precisely a cube of length L generated with periodic boundary conditions. We use the MPB software (*5*) to obtain the eigenfrequencies of the photonic crystal. On the one hand, if a stop band opens between bands N and $N+1$, we expect $N \propto (L/a)^3$, i.e. we expect N to be proportional to the number of scattering units in the primitive cell. On the other hand, for a given spatial resolution δx, the description of the permittivity contains $\mathcal{O}\left(\frac{L}{\delta x}\right)^3$ voxels in the cell, and we need $\mathcal{O}\left(\frac{L}{a}\right)^3$ bands as discussed above. It is clear that a calculation with high resolution and large supercells will be prohibitively expensive both in terms of memory and computer time. In particular, our model network derived from the pattern used in the main text (*1*) does not fit into our available computational facilities. However, a smaller system with $L \simeq 4.34a$ and a resolution of 128^3 voxels fits into the memory and can be solved for 8 different points in the reciprocal space with reasonable computational efforts and a resolution being comparable to the one used in transport calculations. In this case the computation of the first thousand eigenfrequencies for each k point is enough to cover the spectral region of interest.

In order to generate a suitable seed pattern, we obtain a random closed packing of N_S rigid spheres in a periodic cube. We used the software developed by Torquato and co-workers (*2*). Setting a packing fraction $\phi_s \simeq 64\%$, we obtain a random close packed distribution. The centers of the spheres are the seed pattern to generate the random four-valent network as explained in the main text. Setting the appropriate diameter of cylinders linking the nodes of the structure, we obtain a perfectly periodic structure with filling fraction $\phi = 28.14\%$ which is, with high precision, the same as the one used for transport calculations considered in the main text. We notice at this point that the Delaunay tessellation is performed in a $3 \times 3 \times 3$ replica of the

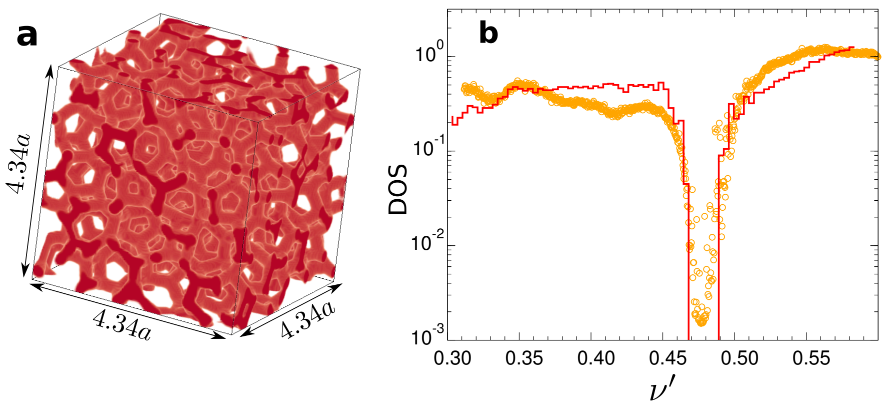

Supplementary Figure 2: (**a**) Network with $n = 3.6$ used to calculate the band structure with the MPB software. (**b**) DOS obtained by Liew et.al. (*4*) (open symbols) compared with a frequency histogram of our calculated band structure (solid line). We note that in ref. (*4*) a denotes the mean spacing between spheres of radius R while here we plot both data sets with $a = 2R$, where R is the diameter of the sphere. Our value of a is smaller by a factor 0.9348 compared to the value used in (*4*).

original seed pattern. Cutting the final structure to the original size of the cube avoids any spurious surface effect in the final network.

In Supplementary Figure 2 we summarize our results for one of the structures. In panel a, we represent the spatial structure of the permittivity as interpreted by the MPB software. Interestingly, using a seed pattern with only a hundred spheres is enough to generate a relatively large network. In panel b, we compare the DOS as presented by Liew et al. (*4*) together with a histogram of all the frequencies given by the MPB software of the structure depicted in panel a (all other parameters kept as in the main text). The dip in the DOS corresponds almost exactly with the exact zero of the frequency histogram. In the MPB we sampled the k space using 8 points distributed along the $\Gamma - R - X - M - \Gamma$ path in the reciprocal space with one interpolating point between the vertices.

Effective refractive index. From DOS calculations we find that the gap center-wavelength λ_G is red-shifted when increasing the volume fraction of the higher refractive index component,

Supplementary Figure 3 (see also ref. (*4*)). In the long wavelength limit $\lambda \gg a$, i.e. for wavelengths large compared to the size of dielectric heterogeneities, the well known Maxwell-Garnet mixing formula can be applied. If, however, heterogeneities are present on length scales comparable to the wavelength, the situation becomes more complicated. Different mixing formulas have been proposed to determine an effective permittivity or effective refractive index. A set of commonly used formulas can be written in a unified way, as follows (for details we refer to the textbook by Sihvola (*7*)):

$$\frac{\varepsilon_{\text{eff}} - \varepsilon_e}{\varepsilon_{\text{eff}} + 2\varepsilon_e + \alpha\left(\varepsilon_{\text{eff}} - \varepsilon_e\right)} = \frac{\phi\left(\varepsilon - \varepsilon_e\right)}{\varepsilon + 2\varepsilon_e + \alpha\left(\varepsilon_{\text{eff}} - \varepsilon_e\right)} \tag{S1}$$

ϕ denotes the filling fraction, ε and ε_e are the dielectric permittivity of the dispersed or structured material and the background environment, respectively, and $n_{\text{eff}} = \sqrt{\varepsilon_{\text{eff}}}$ is the effective refractive index. For our case $\varepsilon = 3.6^2 = 12.96$ for silicon and $\varepsilon_e = 1$ for air. For a given value of α we can calculate ε_{eff} from Eq.(S1) and thus obtain n_{eff} for all possible compositions ϕ. Depending on the choice of the dimensionless parameter α different mixing rules are recovered: ($\alpha = 0$) Maxwell-Garnett, ($\alpha = 1$) Polder-van Santen or Bruggemann and ($\alpha = 2$) Coherent Potential approximation. Here we treat α as an adjustable parameter to obtain a best fit to the $\nu'_{\text{Gap}}(\phi) = a/\lambda_{\text{Gap}}(\phi)$, with $\nu'_{\text{Gap}}(\phi) = \nu'_{\text{Gap}}(0) \times n_{\text{eff}}(\phi)$, Supplementary Figure 3. We find $\alpha \simeq 0.7$ and $\nu'_{\text{Gap}}(0) = 0.34$. Thus for $\phi = 0.28$ we find $n_{\text{eff}} \simeq 1.42$.

Angle Averaged Reflection An important parameter in both standard diffusion theory and self consistent theory of localization is the extrapolation length z_0. If internal reflections are not considered, $z_0\ell = 2\ell/3$ (we set $\ell^* = \ell_s \equiv \ell$). Considering the effect of internal reflection from the effective medium to vacuum leads to a larger z_0 that can be written as

$$z_0 = \frac{2}{3}\frac{1+R}{1-R}, \tag{S2}$$

where R is the angle averaged reflectivity from the effective medium of refractive index n_{eff} to

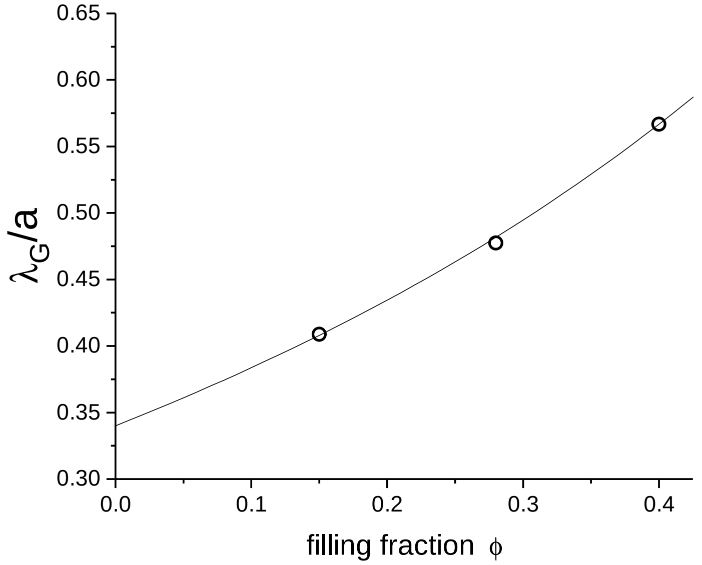

Supplementary Figure 3: Open symbols: Band gap center frequency $\nu'_{\text{Gap}}(\phi) = a/\lambda_{\text{Gap}}(\phi)$ extraced from ref. (*4*) for different silicon filling fractions ϕ. Line: Best fit to $\nu'_{\text{Gap}}(\phi) = \nu'_{\text{Gap}}(0) \times n_{\text{eff}}(\phi)$ with $n_{\text{eff}}(\phi)$ using the unified mixing formula, Eq. S1. Parameters are $\alpha = 0.7$ with $\nu'_{\text{Gap}}(0) = a/\lambda_{\text{Gap}}(0) = 0.34$. For $\phi = 0.28$ we find $n_{\text{eff}} = 1.42$.

the vacuum. Considering a uniform angular distribution of the intensity, R is given by

$$R = \mu_c + \int_{\mu_c}^{1} d\mu R\left(\mu\right), \tag{S3}$$

where $\mu = \cos\left(\theta\right)$ (θ =angle formed by the propagation direction with the direction perpendicular to the slab), $R\left(\mu\right)$ is the polarization averaged reflection at the considered angle, and μ_c is the cosine of the critical angle $\mu_c = \sqrt{1 - 1/n_{\text{eff}}^2}$. $R\left(\mu\right)$ is given by

$$R\left(\mu\right) = \frac{1}{2}\left|\frac{n_{\text{eff}}\mu - \mu_2}{n_{\text{eff}}\mu + \mu_2}\right|^2 + \frac{1}{2}\left|\frac{n_{\text{eff}}\mu_2 - \mu}{n_{\text{eff}}\mu_2 + \mu}\right|^2, \tag{S4}$$

with $\mu_2 = \sqrt{1 + n_{\text{eff}}^2\left(\mu^2 - 1\right)}$ is the cosine of the transmitted wave. In Supplementary Figure 4, the angle and polarization averaged reflection R is plotted as a function of the effective refractive index. The averaged reflection at $n_{\text{eff}} = 1.42$ ($R \simeq 0.734$) is highlighted with a full circle.

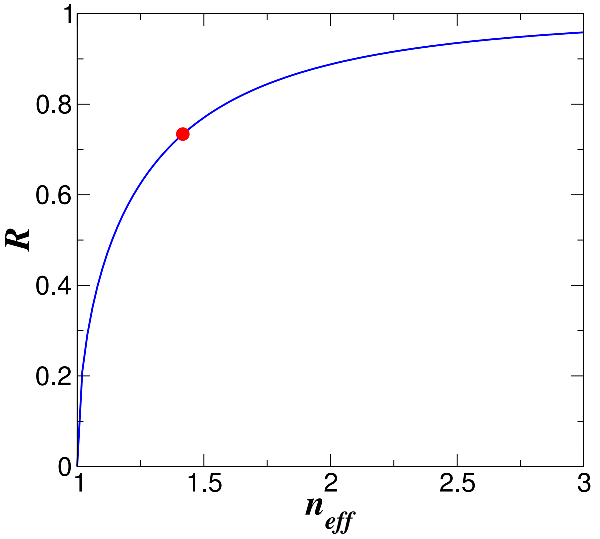

Supplementary Figure 4: Angle and polarization averaged reflectivity from a medium of refractive index n_{eff} to vacuum.

Diffuse transmission and reflection as a function of source position in SC theory. We consider the Green function for the diffusion equation with a position-dependent diffusion coefficient $D(z)$. In this section we normalize all lengths and positions by the mean free path ℓ, and the diffusion coefficient $D\left(z\right)$ by the Boltzmann transport theory diffusion constant D_B.

$$-D\left(z\right)\frac{\partial^2 g\left(z,z'\right)}{\partial z^2}-\frac{\partial D\left(z\right)}{\partial z}\frac{\partial g\left(z,z'\right)}{\partial z}=\delta\left(z-z'\right) \tag{S5}$$

with boundary conditions

$$g\left(z=0,z'\right)=z_0 D\left(z=0\right)\left.\frac{\partial g\left(z,z'\right)}{\partial z}\right|_{z=0^+} \tag{S6a}$$

$$g\left(z=L,z'\right)=-z_0 D\left(z=L\right)\left.\frac{\partial g\left(z,z'\right)}{\partial z}\right|_{z=L^-} \tag{S6b}$$

Assuming that $D\left(z\right)>0$, we can transform Eq.(S5) to

$$-\frac{\partial^2 g\left(z,z'\right)}{\partial z^2}-LN\left(z\right)\frac{\partial g\left(z,z'\right)}{\partial z}=\frac{1}{D\left(z'\right)}\delta\left(z-z'\right), \tag{S7}$$

where

$$LN(z) \equiv \frac{d\left[\ln\left(D(z)\right)\right]}{dz} \tag{S8}$$

For $z < z'$ equation (S7) can be integrated. We have

$$\begin{aligned}\frac{dg\left(z<z',z'\right)}{dz} &= \frac{dg\left(z=0^{+},z'\right)}{dz}\exp\left[-\int_0^z LN\left(x\right)dx\right]\\ &= \frac{dg\left(z=0^{+},z'\right)}{dz}\exp\left[\ln\left(D\left(z=0\right)\right)-\ln\left(D\left(z\right)\right)\right]\\ &= \frac{dg\left(z=0^{+},z'\right)}{dz}\frac{D\left(0\right)}{D\left(z\right)}\end{aligned} \tag{S9}$$

using the boundary condition Eq.(S6a) we have

$$\frac{dg\left(z<z',z'\right)}{dz} = \frac{g\left(0,z'\right)}{z_0}\frac{1}{D\left(z\right)} \tag{S10}$$

and, integrating Eq.(S10),

$$g\left(z<z',z'\right) = g\left(0,z'\right)\left[1+\frac{1}{z_0}\int_0^z \frac{1}{D\left(x\right)}dx\right]. \tag{S11}$$

Analogously, for $z > z'$, and considering the boundary condition given by Eq.(S6b) we have

$$g\left(z>z',z'\right) = g\left(L,z'\right)\left[1+\frac{1}{z_0}\int_z^L \frac{1}{D\left(x\right)}dx\right] \tag{S12}$$

To solve for the Green function Eq.(S5) we have consider the discontinuity in the first derivative of $g\left(z,z'\right)$ at $z \to z'^{\pm}$, i.e.

$$\frac{dg\left(z=z'^{+},z'\right)}{dz} - \frac{dg\left(z=z'^{-},z'\right)}{dz} = \frac{-1}{D\left(z'\right)} \tag{S13}$$

and the continuity of the Green function in one dimension

$$g\left(z=z'^{+},z'\right) = g\left(z=z'^{-},z'\right). \tag{S14}$$

Considering the solutions Eq.(S11,S12) and the previous matching conditions for the Green function Eq.(S13,S14) we have

$$g(0,z') = z_0 \frac{z_0 + \int_{z'}^{L} \frac{1}{D(x)} dx}{2z_0 + \int_0^L \frac{1}{D(x)} dx} \tag{S15a}$$

$$g(L,z') = z_0 \frac{z_0 + \int_0^{z'} \frac{1}{D(x)} dx}{2z_0 + \int_0^L \frac{1}{D(x)} dx} \tag{S15b}$$

If J_d^- is the diffusive flux in $z < z'$ and $J_d^+$is the diffusive flux in $z > z'$ we can define the diffuse transmittance and reflectance $T_{SCT}(z')$ and $R_{SCT}(z')$ resp. as

$$T_{SCT}(z') \equiv J_d^+ = -D(L) \frac{dg(z = L^+, z')}{dz} = \frac{g(z = L^+, z')}{z_0} = \frac{z_0 + \int_0^{z'} \frac{1}{D(x)} dx}{2z_0 + \tilde{L}} \tag{S16a}$$

$$R_{SCT}(z') \equiv -J_d^- = D(0) \frac{dg(z = 0^+, z')}{dz} = \frac{g(z = 0^+, z')}{z_0} = \frac{z_0 + \int_{z'}^{L} \frac{1}{D(x)} dx}{2z_0 + \tilde{L}} \tag{S16b}$$

Where we have defined

$$\tilde{L} \equiv \int_0^L \frac{1}{D(x)} dx \tag{S17}$$

Supplementary References

1. Song, C., Wang, P. & Makse, H. A. A phase diagram for jammed matter. *Nature* **453**, 629 (2008).

2. Skoge, M., Donev, A., Stillinger, F. H. & Torquato, S. Packing hyperspheres in high-dimensional euclidean spaces. *Physical Review E* **74**, 041127 (2006).

3. Froufe-Pérez, L. S. *et al.* Role of short-range order and hyperuniformity in the formation of band gaps in disordered photonic materials. *Physical Review Letters* **117**, 053902 (2016).

4. Liew, S. F. *et al.* Photonic band gaps in three-dimensional network structures with short-range order. *Physical Review A* **84**, 063818 (2011).

5. Johnson, S. & Joannopoulos, J. Block-iterative frequency-domain methods for Maxwell's equations in a planewave basis. *Optics Express* **8**, 173–190 (2001).

6. Florescu, M., Torquato, S. & Steinhardt, P. J. Designer disordered materials with large, complete photonic band gaps. *Proceedings of the National Academy of Sciences* **106**, 20658–20663 (2009).

7. Sihvola, A. H. *Electromagnetic mixing formulas and applications*. 47 (The Institution of Engineering and Technology, London, UK, 1999, 1999).
